# Opportunistic mammography screening provides effective detection rates in a limited resource healthcare system

**DOI:** 10.1186/s12885-015-1419-2

**Published:** 2015-05-15

**Authors:** Yew-Ching Teh, Gie-Hooi Tan, Nur Aishah Taib, Kartini Rahmat, Caroline Judy Westerhout, Farhana Fadzli, Mee-Hoong See, Suniza Jamaris, Cheng-Har Yip

**Affiliations:** 1 Gleneagles Medical Centre, Penang, Malaysia; 2 Department of Surgery, University Malaya Medical Centre, Kuala Lumpur, Malaysia; 3 Department of Biomedical Imaging, University Malaya Medical Centre, Kuala Lumpur, Malaysia

**Keywords:** Opportunistic screening mammography, Performance indicators, Breast cancer, Low and middle income country

## Abstract

**Background:**

Breast cancer is the leading cause of cancer deaths in women world-wide. In low and middle income countries, where there are no population-based mammographic screening programmes, late presentation is common, and because of inadequate access to optimal treatment, survival rates are poor. Mammographic screening is well-studied in high-income countries in western populations, and because it has been shown to reduce breast cancer mortality, it has become part of the healthcare systems in such countries. However the performance of mammographic screening in a developing country is largely unknown.

This study aims to evaluate the performance of mammographic screening in Malaysia, a middle income country, and to compare the stage and surgical treatment of screen-detected and symptomatic breast cancer.

**Methods:**

A retrospective review of 2510 mammograms performed from Jan to Dec 2010 in a tertiary medical centre is carried out. The three groups identified are the routine (opportunistic) screening group, the targeted (high risk) screening group and the diagnostic group. The performance indicators of each group is calculated, and stage at presentation and treatment between the screening and diagnostic group is analyzed.

**Results:**

The cancer detection rate in the opportunistic screening group, targeted screening group, and the symptomatic group is 0.5 %, 1.25 % and 26 % respectively. The proportion of ductal carcinoma in situ is 23.1 % in the two screening groups compared to only 2.5 % in the diagnostic group. Among the opportunistic screening group, the cancer detection rate was 0.2 % in women below 50 years old compared to 0.65 % in women 50 years and above. The performance indicators are within international standards. Early-staged breast cancer (Stage 0–2) were 84.6 % in the screening groups compared to 61.1 % in the diagnostic group.

**Conclusion:**

From the results, in a setting with resource constraints, targeted screening of high risk individuals will give a higher yield, and if more resources are available, population-based screening of women 50 and above is effective.

Opportunistic mammographic screening is feasible and effective in a middle income country with performance indicators within international standards. Waiting until women are symptomatic will lead to more advanced cancers.

## Background

Breast cancer is the leading cause of cancer deaths in women worldwide. The two main determinants of survival are early detection and optimal treatment. In low and middle income countries (LMICs), late presentation of breast cancer is common. While geographical isolation and poverty may lead to delayed presentation, psychosocial and cultural beliefs are also major barriers [1]. The three methods of early detection are breast self-examination (BSE), clinical breast examination (CBE) and mammography. While BSE and CBE can lead to downstaging of symptomatic disease, screening for asymptomatic disease by mammography will allow for detection of breast cancer in the earliest stage where cure is possible [2].

There is no population based mammographic screening programme in most LMICs including Malaysia, because such a programme would require not only the facilities and manpower for the process of screening, but also a robust and equitable healthcare system that can provide for the diagnosis, treatment and follow-up of women with abnormalities diagnosed on screening. However, in a country like Malaysia, which is in transition from a developing to a developed country, women especially in the urban areas in Malaysia, are becoming more educated and with information gleaned from the media about the increasing incidence of breast cancer, and the importance of early detection, more women are coming forward for screening mammography. University Malaya Medical Centre (UMMC) is a public hospital in an urban area in Malaysia, and since 1993, has a mammography service which provides opportunistic and targeted mammographic screening, together with diagnostic mammograms at request of doctors from the Breast Clinic, Primary Medicine clinic as well as from the Gynaecology Clinic. The objective of this study is to evaluate the performance of opportunistic screening mammography in a fully equipped tertiary medical centre with a breast unit and to compare the stage and treatment of screen-detected to that of symptomatic breast cancers.

## Methods

A retrospective study of 2510 consecutive full film digital mammograms (FFDM) performed at the University Malaya Medical Centre (UMMC), a tertiery teaching hospital in an urban setting, from January to December 2010 were reviewed. This study was approved by the Ethical Review Committee of UMMC. As is the current practice in the breast imaging unit of the hospital, two views (medio-lateral and cranio-caudal) are carried out and the mammograms are reported immediately by trained breast radiologists. Adjunct ultrasound is carried out at the same time when deemed necessary, usually for dense breasts or for further evaluation of any mammographic abnormality. The reason for the mammogram is stated in the mammogram request form, and this information is available to the radiologist. 2178 mammograms were performed on asymptomatic women while 332 (13.2 %) were diagnostic mammograms. In the former group, 1938 (77.2 %) were routine screening mammograms (defined as 40 years and above with an average risk for developing breast cancer), while 240 (9.6 %) were targeted mammographic screening in a group of women at higher risk for developing breast cancer (defined as a positive family history, previous biopsy showing atypical ductal hyperplasia, or on hormone replacement therapy). In the diagnostic group, 20 had already had a biopsy showing a malignancy before mammography was carried out, and these were excluded; leaving 312 for analysis. The ages of the women, Breast Imaging Reporting and Data System (BI-RADS) assessment category (Table 1) [3], breast density composition, adjunct ultrasound, type of biopsy and histopathological results were retrieved from the computerized imaging system, imaging reports and hospital records. The data was analyzed using SPSS Statistics Version 22. The stage of the cancer and surgical treatment in the screening groups were compared with the diagnostic group. The routine screening group (1938 women) was used as a surrogate for the opportunistic screening group and separately analyzed.

**Table 1:** BIRADS classification

BIRADS	Definition	Opportunistic MMG screening		Targeted MMG screening		Diagnostic MMG	
		No	%	No	%	No	%
0	Incomplete	7	0.4	1	0.4	0	
1	Negative	887	45.8	102	42.5	47	15.1
2	Benign findings	911	47	114	47.5	103	33.0
3	Probably benign abnormality	93	4.8	11	4.6	49	15.7
4	Suspicious abnormality	35	1.8	11	4.6	51	16.3
5	Highly suggestive of malignancy	5	0.2	1	0.4	62	19.9
	Total	1938	100	240	100	312	100

## Results

In the targeted screening group, 219 had a family history of breast cancer (65 % were in first degree relatives), 13 were on hormone replacement therapy, while 8 had a previous excision biopsy showing atypical ductal hyperplasia. In the diagnostic group, the commonest symptom was a breast lump, and the median duration of symptoms was 6 months. Table 1 shows the BI-RADS assessment category in the three groups of women presenting for mammography. In the screening groups, the majority of women were classified as BIRADS 1 and 2 which do not require any further follow-up. Table 2 shows that the mean age was similar in the three groups. The majority of patients were Chinese, which is consistent with the higher incidence of breast cancer in the Chinese population in Malaysia.

**Table 2:** Demographics and performance of screening and diagnostic mammogram

	Screening mammogram				Diagnostic mammogram	
	Opportunistic screening mammogram		Targeted screening mammogram			
	No	%	No	%	No	%
Mean age (years)	55.6		56.4		54.2	
Race						
Chinese	946	48.8	130	54.2	121	38.8
Malays	428	22.1	63	26.3	71	22.8
Indians	529	27.3	43	17.9	115	36.9
Others	35	1.8	4	1.7	5	1.6
Total	1938	100	240	100	312	100
Cancer detection rate (invasive and in-situ)	0.52		1.25		26.0 %	
Invasive carcinoma	8		2		79	
DCIS	2 (20 %)		1 (33.3 %)		2 (2.5 %)	
No of biopsies	44		13		149	
Biopsy rate	2.3 %		5.4 %		47.8 %	
PPV for abnormal mammogram (BIRADS 3–5)	7.5 %		13 %		63.4 %	
PPV for suspicious mammogram (BIRADS 4–5)	25 %		25 %		71.7 %	
PPV for biopsies	22.7 %		23 %		54.3 %	

In the diagnostic group, 81 of the 312 women (26 %) were confirmed to have invasive and non-invasive breast cancer compared to only 10 out of 1938 (0.52 %) in the opportunistic screening group. In the 240 women with an elevated risk of breast cancer (family history and atypical ductal hyperplasia), 3 cancers were found giving a pick-up rate of 1.25 %.

It is interesting to note that in the diagnostic group, there were only two women with ductal carcinoma-in-situ (DCIS) compared to 79 with invasive cancer (DCIS rate of 2.5 %). In the two screening groups, there were three in-situ cancers compared to 10 invasive cancers (DCIS rate of 23.1 %) The biopsy rate in the opportunistic screening group was 2.3 %, while it was 5.4 % in the targeted screening group. This is compared to the diagnostic group which had a biopsy rate of 47.8 %. The positive predictive value (PPV) for biopsies was 22.7 % and 23 % in the opportunistic screening group and targeted screening group respectively. As expected, PPV for biopsies was very high (54.3 %) in the diagnostic group. BI-RADS 4–5 is classified as a suspicious examination with positive finding which requires tissue diagnosis for confirmation, whereas BI-RADS 3 category are “probably benign findings” which require a close follow-up. In the two screening groups, one out of the 125 BIRADS 3 (0.8 %), 7 out of 57 with a report of BIRADS 4 (12.2 %) and 5 out of 7 BIRADS 5 (71.4 %) were malignant. The PPV for abnormal mammogram (BI-RADS 3–5) was 7.5 % in the opportunistic screening group and double that for the targeted screening group. PPV for abnormal mammogram in the diagnostic group was very high (63.2 %). When we consider BI-RADS 4–5 as the positive group, the PPV increased to 25 % in both the opportunistic and targeted screening groups, while it was 71.7 % in the diagnostic group.

When the stage at diagnosis in the screening groups was compared with the diagnostic group, it was shown that 84.6 % breast cancer diagnosed by screening was in the early stages (Stage 0–2) compared to 61.1 % in the diagnostic group. However, of those who had surgery, 76.9 % of the screening group had mastectomy compared with 77.3 % in the diagnostic group, showing that there was no difference in the mastectomy rate, whether screen detected or detected with symptoms (Table 3).

**Table 3:** Stage of Breast Cancer at Diagnosis and Surgical Treatment

Stage at diagnosis	Screening mammogram (targeted and opportunistic)	Diagnostic mammogram	P value
0	3 (23.0 %)	2 (2.6 %)	0.00*
1	4 (30.8 %)	18 (23.4 %)	
2	4 (30.8 %)	27 (35.1 %)	
3	2 (15.4 %)	14 (18.2 %)	
4		16 (20.7 %)	
Unstaged	0	4	
Type of Surgery			
Mastectomy	10 (76.9 %)	51 (77.3 %)	0.97
Breast conserving surgery	3 (23.1 %)	15 (22.7 %)	
No surgery done	0	15	

*Significant

The effect of age on performance in the opportunistic screening group was separately analyzed (Table 4) in this group, 25 % were less than 50 years old. As expected, breast density was significantly higher (p = 0.00) in the < 50 age group (22.5 % dense and 66.3 % moderately dense) compared to the 50 and above age group (12.4 % dense and 60.5 % moderately dense). Supplementary ultrasound was done in 31.7 % of women undergoing routine mammography and it was more likely to be carried out in the <50 age group (p = 0.01) The cancer detection rate was only 0.2 % in women < 50 years old compared to 0.62 % in women 50 and above. The biopsy rate was significantly higher in the < 50 age group, while the positive predictive value (PPV) for biopsies were significantly higher in the 50 years and above age group.

**Table 4:** Effect of age on performance of opportunistic mammogram screening

	Less than 50 years old	50 years and above	All age groups	P value
Number	484 (25 %)	1454 (75 %)	1938	
Cancer detection rate	0.20 %	0.62 %	0.52 %	0.2
Dense breasts	22.5 %	12.4 %	15 %	0.00*
Moderately dense breasts	66.3 %	60.5 %	62.4 %	
Adjunct ultrasound done	42.7 %	28.1 %	31.7 %	0.00*
BIRADS 3–5 (abnormal mammogram)	40 (8.2 %)	93 (6.3 %)	133 (6.8 %)	0.2
BIRADS 4–5 (suspicious mammogram)	10 (2.1 %)	30 (2.1 %)	40 (2.1 %)	
Biopsy rate	3.7 %	1.8 %	2.3 %	0.028*
PPV for abnormal mammogram	2.5 %	9.6 %	7.5 %	0.1
PPV for suspicious mammogram	10 %	30 %	25 %	0.2
PPV for biopsies	5.5 %	34.6 %	22.7 %	0.01*

*Significant

## Discussion

A meta-analysis of 11 randomized controlled trials (RCTs) on mammographic breast screening showed a 20 % reduction in breast cancer mortality in women invited for screening with 13 years of follow-up, particularly in the 50–69 year age group [4]. However the question of overdiagnosis and overtreatment has been debated in recent years, and it has been recommended that women should be fully informed about the harms and benefits before they decide whether to screen or not [5]. In Asian countries, there have been no RCTs on population-based mammographic screening, except for a pilot prevalence screening programme in Singapore [6]. The performance of opportunistic screening programmes have been reported in Japan [7] and Hong Kong [8]. However these are high income countries in Asia. Very little data on opportunistic mammographic screening is available in LMICs in Asia.

Population-based cancer screening is distinguished from opportunistic screening on the basis of how invitations to screening are extended. Population-based screening is issued from population-based registers, while opportunistic screening depends on an asymptomatic individual’s personal decision whether to screen or not, or based on advice from an encounter with a health professional. Population-based programs focus on reducing mortality and morbidity from breast cancer at the level of the population, while opportunistic screening may not have all the hallmarks of a population-based program, namely, eligibility requirements, quality assurance, follow-up and evaluation [9]. A study in Switzerland showed that population-based mammographic screening was as effective as opportunistic screening [10] while another study in Denmark showed that population-based screening had considerably higher sensitivity than opportunistic screening, while the specificity was similar in the two settings [11].

Rigorous, high quality screening, diagnosis and treatment are crucial in attaining the goal of reducing breast cancer mortality rates through screening. The programme needs to be audited to ensure that performance indicators are met [12]. Unfortunately not all the elements required for a population-based mammographic screening programme, namely manpower, quality assurance and optimal access to treatment, are available in low and middle income countries. In such countries, several opportunistic programmes are available through hospitals, with several funded by non-governmental organizations. However most of these programmes are not evaluated. This study in a tertiary hospital has the elements required for a high-quality programme, with a multidisciplinary breast team in place since 1993.

The majority of women were Chinese which reflects the location of UMMC in an urban mainly Chinese neighbourhood. The mean age of 55.6 years was typical of the age group eligible for mammographic screening which is between 40 and 70. Because the most prevalent age group for breast cancer in Malaysia is 40–49 years old [13], this age group is offered mammographic screening if they wish to do so, and comprises 25 % of opportunistic mammographic screening group. The cancer detection rate in this group was 0.5 %, which is consistent with what was reported in opportunistic screening programmes in Japan and Hong Kong [7, 8], where the age standardized incidence rate of breast cancer is similar. The pilot population based mammographic screening programme in Singapore reported a cancer detection rate of 0.48 % [6]. As expected the cancer detection rate was lower than that reported in high incidence countries like USA and UK at first mammogram screening (0.8 and 1.0 % respectively) [14].

In the group of women with a risk factor, such as family history, hormone replacement therapy, and atypical ductal hyperplasia (targeted screening) the cancer detection rate was more than doubled (1.25 %), as expected, as their risk of developing breast cancer is higher.

What is striking in the two screening groups is that the proportion of DCIS (ductal carcinoma in situ) was 23.1 %, compared to only 2.5 % in the diagnostic group. The DCIS rate in the study in Singapore was 26 % [6], 30 % in Japan [7] and 28 % in Hong Kong [8]. In contrast the DCIS rates in high-incidence countries like UK and USA are much lower (8.3 % and 12.3 %) [14]. The proportion of DCIS in this study is also well above the minimum standard of 10 % set by the IARC [12]. The higher rate of screen detected DCIS in Asians have also been noted in other studies, and is postulated to be due to a different pattern of growth in Asian women [15].

31.5 % of women undergoing routine mammography screening had ultrasound done, especially in women below 50 years old. In an opportunistic setting as practiced by UMMC, mammograms are immediately read and any further imaging is carried out at the same visit. It is also noted that Asian women, especially of Chinese ethnicity, have denser breasts [16] and supplementary ultrasound in such cases would be able to identify any abnormalities missed on mammography alone [17]. 77.4 % of women had dense or moderately dense breasts in this study and mammography density was higher in younger women. In population-based screening, the mammogram is reported later and if any additional imaging is to be carried out, the woman is recalled for further tests. The percentage of recall should be less than 7 % according to the performance indicators set up by IARC [12], hence a supplementary ultrasound of 31.5 % appears excessive, but opportunistic screening is very much at the individual level rather than at the population level.

For a screening programme to be cost-effective, recall rates and biopsy rates should be low, and PPV should be high. The PPV of an abnormal mammogram in the opportunistic screening arm of this study was 7.5 %, biopsy rate was 2.3 %, and the PPV for biopsies was 22.7 %. This is acceptable by international standards [18] and similar to the reported rates in Japan [7] and Hong Kong [8]. Biopsy rates are higher in the targeted screening group, which also has a higher PPV for biopsies and for abnormal mammograms.

Although the stage of the cancer in the screening arms was significantly earlier compared to the stage in the diagnostic arm (84.6 % Stage 0–2 compared with 61.1 %, p = 0.00), the mastectomy rates between the screening and diagnostic groups were not different. It has been reported that Asian patients are more likely to choose mastectomy for a variety of reasons, the most important of which is that they feel safer, and also because they do not want radiotherapy [19]. It is also interesting to note that in the diagnostic group, 4 women (4.9 %) defaulted any further management and were not staged, while 15 women in the diagnostic group did not have any surgery carried out, either because they refused or were metastatic at diagnosis. Delayed presentation and defaulting treatment has been described in LMICs [1, 20].

The cancer detection rate was lower in the age group below 50 years old (0.2 %) compared with the 50 years and above age group (0.62 %). This differs from the opportunistic screening programme in Japan and Hong Kong where cancer detection rates were higher in women less than 50 years old [7, 8] but similar to a study in USA where cancer detection rates were higher in the older age group [21]. Since the prevalent age group for breast cancer in Malaysia is the 40–49 year age group, it is difficult to understand the lower cancer detection rate in the younger women; however it could be that there were false negatives in this group since the breasts are dense and cancers may be missed. While the biopsy rate in women below 50 years is higher, the PPV for positive biopsies was much lower in this age group.

The limitation of this study is that it is a single snapshot of the mammography service in UMMC over a one-year period, and the false negative rate and interval cancer rate is unknown. However the opportunistic screening mammogram group can be a surrogate of whether a population-based programme is feasible but only if the similar facilities are available. Depending on the resources available, a targeted screening programme (for women with increased risk) with its higher cancer detection rate should be carried out initially and once resources become more available, routine screening for women 50 years and above. In Malaysia, it is not likely to be cost-effective to routinely screen women below the age of 50 years, because of the low cancer detection rate, high biopsy rate, and the PPV for biopsies is below the standards set by IARC. However it is noted that the current policy by the Ministry of Health in Malaysia to offer free mammographic screening to women who are at higher risk of breast cancer based on known risk factors may miss a significant number of cancers as 10 out of the 13 cancers found (76.9 %) were from women with no risk factors.

## Conclusions

Based on this study, the performance of mammographic screening is within acceptable international standards. Screening a high risk group would give the best cancer detection rate, with acceptable biopsy rate, PPV for abnormal mammogram and biopsies. With more resources, screening can be extended to all women 50 years and above, as the cancer detection rate and acceptable performance indicators make it feasible. Waiting for women to become symptomatic will lead more advanced cancers, which will require more resources for treatment.
